# Scyphiphin C, a New Iridoid from *Scyphiphora hydrophyllacea*

**DOI:** 10.3390/molecules15042473

**Published:** 2010-04-08

**Authors:** Cui-Lian Feng, Ming-Fu Gong, Yan-Bo Zeng, Hao-Fu Dai, Wen-Li Mei

**Affiliations:** 1 Key Laboratory of Tropical Crop Biotechnology, Ministry of Agriculture, Institute of Tropical Bioscience and Biotechnology, Chinese Academy of Tropical Agricultural Sciences, Haikou 571101, Hainan, China; E-Mails: fengcuilian@126.com (C.-L.F.); zengyanbo@163.com (Y.-B.Z.); hfdai2001@yahoo.com.cn (H.-F.D.); 2 Key Laboratory of Protection & Utilization of Biological Resources in Tarim Basin of Xinjiang Production Construction Corps, College of Life Science, Tarim University, Alar 843300, Xingjiang, China; E-Mail: gongmingfu98@163.com (M.-F.G.)

**Keywords:** *Scyphiphora hydrophyllacea*, scyphiphin C, mangrove plant, iridoid

## Abstract

Chemical investigation of the ethanol extract of the aerial parts of *Scyphiphora hydrophyllacea* Gaertn. collected in Hainan Province of China resulted in the isolation of a new iridoid, scyphiphin C (**1**) and a known iridoid glycoside, shanzhiside methyl ester (**2**). Their structures were elucidated by a study of their physical and spectral data.

## 1. Introduction

**Figure 1:**
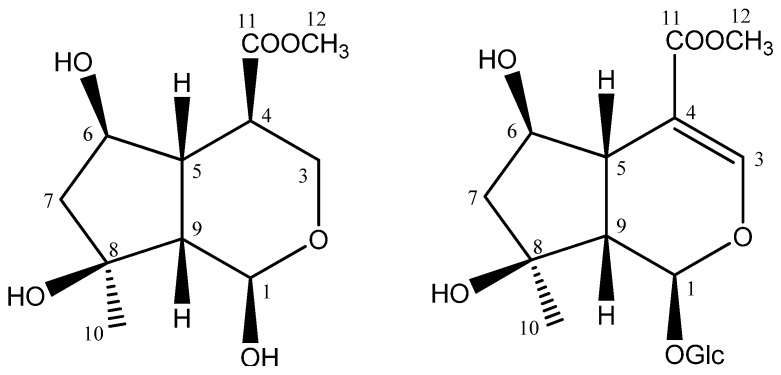
Structures of compounds **1 **and **2**.

Mangrove plants are groups of trees and shrubs growing along seashores in tropical and subtropical areas, which contain plenty of new and bioactive secondary metabolites due to their special eco-environment [1,2,3]. *Scyphiphora hydrophyllacea* Gaertn. F. (Rubiaceae), one of the mangrove plants, is distributed from south to southeast Asia, Caroline Islands, Australia, and New Caledonia [4]. Our previously phytochemical studies showed that flavonoids and terpenoids, especially iridoids, were major constituents of *S*. *hydrophyllacea* [5,6,7,8]. In our continuous search for structurally unique iridoids from this plant, a new iridoid, scyphiphin C (**1**) and a known iridoid glycoside, shanzhiside methyl ester (**2**) (Figure 1), were isolated from the 95% ethanol extract of the aerial parts of this plant. In this paper, we present the isolation and structural characterization of this new iridoid on the basis of the interpretation of spectral data, including 1D and 2D NMR data.

## 2. Results and Discussion

**Figure 2:**
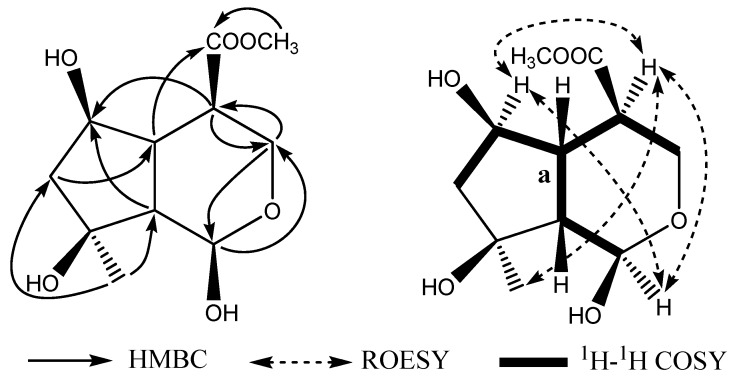
Key HMBC, ROESY and ^1^H-^1^H COSY correlations of compound **1**.

Compound **1**, 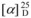 - 13.4 (*c* 0.01, MeOH), was obtained as white amorphous powder. The molecular formula of **1** was established as C_11_H_18_O_6_ with three degrees of unsaturation according to the high-resolution ESI-MS data at *m/z* 269.0998 (calcd. 269.1001 for C_11_H_18_O_6_Na, [M+Na]^+^), which was also supported by ^13^C-NMR and DEPT spectral data. The ^13^C-NMR spectrum (DEPT) showed two quaternary carbons (*δ*_C_ 174.4, 78.6), five methines (*δ*_C_ 94.5, 72.2, 50.7, 48.9, 48.6), two methylenes (*δ*_C_ 65.2, 49.0), and two methyls (*δ*_C_ 52.1, 24.2). The ^1^H-NMR showed a signal characteristic of an iridoid with a proton at *δ*_H_ 4.99 (1H, d, *J* = 9.0 Hz). Interpretation of 2D NMR data especially HMQC correlations and^ 1^H-^1^H COSY correlations of H-4 with H-3 and H-5, H-6 with H-5 and H-7, H-9 with H-1 and H-5, allowed us to construct the partial structure **a** (Table 1 and Figure 2). The ^1^H- and ^13^C- NMR spectroscopic data of **1** were similar to those of aglycone moiety of shanzhigenin methyl ester [9,10,11], the major differences in the ^13^C-NMR spectral data were the presence of a methylene attributed to the 3-position and a methine attributed to the 4-position in **1** instead of the two olefinic carbons seen in shanzhigenin methyl ester. Their chemical shifts were shifted upfield to *δ*_C_ 65.2 (C-3) and 50.7 (C-4), respectively. C-1 was connected with C-3 through an oxygen atom on the basis of the chemical shifts of C-1 (*δ*_C_94.5) and C-3 (*δ*_C_ 65.2), which was also confirmed by the HMBC correlations from H-3 to C-1 and H-1 to C-3. The remaining fragment was confirmed to be identical to that of shanzhigenin methyl ester by analysis of HMQC, HMBC and ^1^H-^1^H COSY spectra. Since the stereochemistry of the three asymmetric centers (C-1, C-5, and C-9) was the same in practically all iridoids identified hitherto [12], 1-OH, H-5, and H-9 were assigned a *β-*orientation in the iridoid skeleton. In the ROESY spectrum (Figure 2), the cross peaks from H-1 to H-4 and H-6 revealed that the relative configuration of H-4 and H-6 were in *α*-orientation. The stereo configuration of Me-10 was determined as *α-*orientation on the basis of the ROESY correlation of Me-10 with H-4. Thus, the structure of compound **1** was identified to have the structure shown in Figure 1, and was named scyphiphin C.

Compound **2 **was determined as shanzhigenin methyl ester by comparison of the ^1^H- and ^13^C-NMR spectral data of **2** with those reported in the literature [9,10,11].

## 3. Experimental

### 3.1. General

Optical rotation was recorded using a Rudolph Autopol III polarimeter (Rudolph Research Analytical, Hackettstown, NJ, USA). The UV spectra were measured on a Shimadzu UV-2550 spectrometer. The IR spectra were obtained as KBr pellets on a Nicolet 380 FT-IR instrument. The NMR spectra were recorded on a Bruker AV-400 spectrometer, using TMS as an internal standard. The HRESIMS spectra were measured with an API QSTAR Pulsar mass spectrometer. Column chromatography was performed with silica gel (Marine Chemical Industry Factory, Qingdao, China) and Sephadex LH-20 (Merck). TLC was preformed with silica gel GF254 (Marine Chemical Industry Factory, Qingdao, China).

**Table 1:** ^1^H- and ^13^C-NMR data of **1** in CD_3_OD. (^1^H- at 400 and ^13^C- at 100 MHz; *J* in Hz).

No.	*δ_C_*	*δ_H_*
1	94.5 ( *d*)	4.99 (1H, *d*, 9.0 Hz)
3	65.2 ( *t*)	3.22 (1H, *t*, 11.8 Hz, H-3*a*)
		3.79 (1H, *ddd*, 0.8, 6.1, 12.2 Hz, H-3*b*)
4	50.7 ( *d*)	2.06 (1H, *m*)
5	48.9 ( *d*)	2.89 (1H, *m*)
6	72.2 ( *d*)	4.42 (1H, *dt*, 3.5, 9.0 Hz)
7	49.0 ( *t*)	2.23 (1H, *dd*, 9.4, 15.1 Hz, H-7*a*)
		1.68 (1H, *dq*, 1.7, 15.0 Hz, H-7*b*)
8	78.6 ( *s*)	
9	48.6 ( *d*)	2.59 (1H, *dd*, 5.6, 9.0 Hz)
10	24.2 ( *q*)	1.15 (3H, *s*)
11	174.4 (s)	
12	52.1 ( *q*)	3.68 (3H, *s*)

### 3.2. Plant Material

The aerial parts of *Scyphiphora hydrophyllacea*Gaertn. F. were collected in Wenchang county (Nov. 2004) in Hainan Province (P.R. China). It was identified by Associate Prof. Zheng-fu Dai of the Institute of Tropical Bioscience and Biotechnology, Chinese Academy of Tropical Agricultural Sciences, where a voucher specimen (SH20051112) is deposited.

### 3.3. Extraction and isolation

The dried, milled aerial parts of *Scyphiphora hydrophyllacea* Gaertn. F. (17.6 kg) were exhaustively extracted with 95 % EtOH (3 × 30 L) at room temperature. After evaporation, the residue was suspended in H_2_O and partitioned with light petroleum to give a light petroleum fraction (687.0 g). The H_2_O part was applied to a D101 reticular resin column eluted with H_2_O and MeOH. The H_2_O eluent was not further fractionated because the major components were sugars. The MeOH eluent was concentrated *in vacuo* to give a residue (421.0 g), which was chromatographed on a silica gel column (200-300 mesh) with CHCl_3_-MeOH [50:1 (2.6 L), 20:1 (21.5 L), 10:1 (17.5 L), 5:1 (21.5 L), 2:1 (21.0 L)] to give 26 fractions. Fraction 16 (5.02 g) was subjected to column chromatography over silica gel eluted with light petroleum-EtOAc (4:6) to afford nine further fractions. Sub-fraction 4 (624.2 mg) was fractionated by column chromatography (Sephadex LH-20) eluted with 95 % EtOH and then rechromatographed on a silica-gel column with light petroleum-EtOAc (3:7) to afford compound **1** (18.7 mg). Fraction 18 (10.06 g) was subjected to vacuum liquid column chromatography over RP-18 eluted with MeOH-H_2_O [1:9 (0.5 L), 2:8 (0.5 L), 3:7 (0.5 L), 4:6 (0.5 L), 1:1 (0.5 L), 6:4 (0.5 L), 8:2 (0.5 L), 0:1 (0.5 L)] gradually to afford eight further fractions. Sub-fraction 1 (2.18 g) was purified by silica gel CC eluted with CHCl_3_-MeOH (9:1) to afford compound **2 **(290.2 mg).

*Scyphiphin C* (**1**): White amorphous powder, 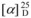 = - 13.4 (*c* 0.01, MeOH). IR (KBr): n = 3875, 3858, 3605, 3308, 2941, 2247, 1482, 865 cm^-1^. HR-ESI-MS (positive): *m/z* = 269.0998 (calcd. 269.1001 for C_11_H_18_O_6_Na, [M+Na]^+^). ^1^H and ^13^C-NMR: see Table 1.

*Shanzhigenin methyl ester* (**2**): White amorphous powder, 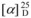 = - 380.0 (*c* = 0.50, MeOH). IR (KBr): n = 3922, 3886, 3779, 3654, 3700, 3543, 3112, 3081, 2326, 1583, 891 cm^-1^. HR-ESI-MS (positive): *m/z* = 429.1380 (calcd. 429.1372 for C_17_H_26_O_11_Na, [M + Na]^+^).

## 4. Conclusions

As a part of our chemical investigation on *Scyphiphora hydrophyllacea*Gaertn., a new iridoid scyphiphin C (**1**) and a known iridoid glucoside shanzhigenin methyl ester (**2**) were isolated. Their structures were established on the basis of spectroscopic evidence.
